# A Single-Stage Medial Opening Wedge High Tibial Osteotomy for Varus Alignment Correction With Revision Arthroscopic Anterior Cruciate Ligament (ACL) Reconstruction

**DOI:** 10.7759/cureus.55992

**Published:** 2024-03-11

**Authors:** Dhruva Angachekar, Sreedhar Archik, Abhay Narvekar, Abhishek Kulkarni, Shivam Patel

**Affiliations:** 1 Orthopaedic Surgery, Paramount Hospital and ICCU, Mumbai, IND; 2 Orthopaedics and Trauma, Gleneagles Hospital, Mumbai, IND; 3 Sports Medicine, P.D. (Parmanand Deepchand) Hinduja National Hospital and Medical Research Centre, Mumbai, IND; 4 Orthopaedics and Trauma, Pravara institute of Medical Sciences, Loni, IND; 5 Orthopaedics, Dr. KNS (Kailash Narayan Singh) Memorial Institute of Medical Sciences, Barabanki, IND

**Keywords:** mowhto, acl revision, anterior cruciate ligament reconstruction (aclr), medial open wedge osteotomy, genu varus, anterior cruciate ligament tear

## Abstract

Anterior cruciate ligament (ACL) injuries are a common clinical entity among people involved in contact sports activities. With the number of primary ACL reconstructions increasing, there has been a proportional increase in the revision of failed ACL reconstruction surgeries. As our understanding of knee kinematics improves over time, there has been evidence that alignment of the lower limb weight-bearing axis also plays an important part in ACL functioning. Medial opening wedge high tibial osteotomy (MOWHTO) is one such procedure that has been used extensively worldwide to correct the varus lower limb alignment. This procedure is usually reserved for young active patients with varus lower limb weight-bearing alignment. The technical dilemma for the surgeon arises when there is a need to revise a failed ACL reconstruction while at the same time correcting the axis malalignment. The general dictum says that alignment correction is done first followed by ligament reconstruction in a dual-stage procedure. However, single-stage surgery is possible in certain indications. In this case report, we present the case of a 31-year-old male involved in recreational sports who sustained a repeat ACL tear five years post the index surgery. He also had a significant varus alignment of the lower limb weight-bearing axis which was considered to be one of the causes of index surgery failure. In this report, we would like to highlight the problems we encountered in a single-stage procedure and certain surgical facets of a single-stage alignment surgery with arthroscopic revision ACL reconstruction.

## Introduction

The goals of anterior cruciate ligament (ACL) reconstruction (ACLR) are to stabilize the knee, stop further injuries, and enable patients to resume their pre-injury level of activity [1]. There has been an increasing trend in the number of arthroscopic reconstruction surgeries being carried out for ACL injuries [2]. With the increase in the number of primary arthroscopic ACL reconstructions, there has been a simultaneous increase in the number of revision ACLR surgeries [3]. The Danish registry has an adult ACLR revision rate of 4.1% after five years, whereas community registries in the United States and Norway record revision rates ranging from 0.9% to 1.5% [4,5]. Nagaraj et al., in their study, similarly found the revision ACL rates to be 4% in the Indian population [6]. According to an extensive meta-analysis, autograft ACLR failure rates are approximately 2.8% [7].

High tibial osteotomy (HTO) is commonly used to treat young, active patients with medial tibiofemoral osteoarthrosis (OA) with varus deformity [8,9]. Numerous procedures, such as dome osteotomy, progressive callus distraction, opening wedge HTO (OWHTO), closing wedge HTO (CWHTO), and "en chevron" osteotomy, can be used to perform an HTO [10]. The most popular procedures are OWHTO and CWHTO, yet there is no proof that one technique is better than the other [11]. Another significant factor in the varus knees lacking in ACL is the posterior tibial slope (PTS) [9]. Researchers have shown that lowering the PTS to normal levels helps decrease anterior tibial translation in knees with ACL deficiency (normal value in the lateral plateau (6-7°) and medial plateau (9-11°)) [12,13]. Depending on the gap height during OWHTO, PTS may be changed [14].

The combination of HTO with ACL reconstruction surgery was first described by O’Neill and James in 1992 [15]. Since then, there have been many studies to evaluate the effectiveness of a single-stage arthroscopic ACL reconstruction with HTO for alignment correction [9,16-18]. In this case report, we try to address the technical points and problems that we encountered in a single-stage revision arthroscopic ACL reconstruction with medial OWHTO (MOWHTO).

## Case presentation

A 31-year-old male patient presented with complaints of instability and pain in his left knee. He had a twisting injury to the left knee while playing casual football two weeks prior to presentation with immediate swelling and inability to flex his left knee. He took diclofenac sodium tablets for pain relief along with local site icing. He was non-weight bearing on the affected leg for five days after which he started to bend his knee and gradually bear weight. His history included arthroscopic ACLR of the left knee seven years prior to this injury. He had sustained a full-thickness ACL tear while playing football which was reconstructed using hamstring autograft. He completed a post-surgery physiotherapy protocol of six months after which he stopped doing the rehab, and continued to play football intermittently as a hobby.

**Figure 1:**
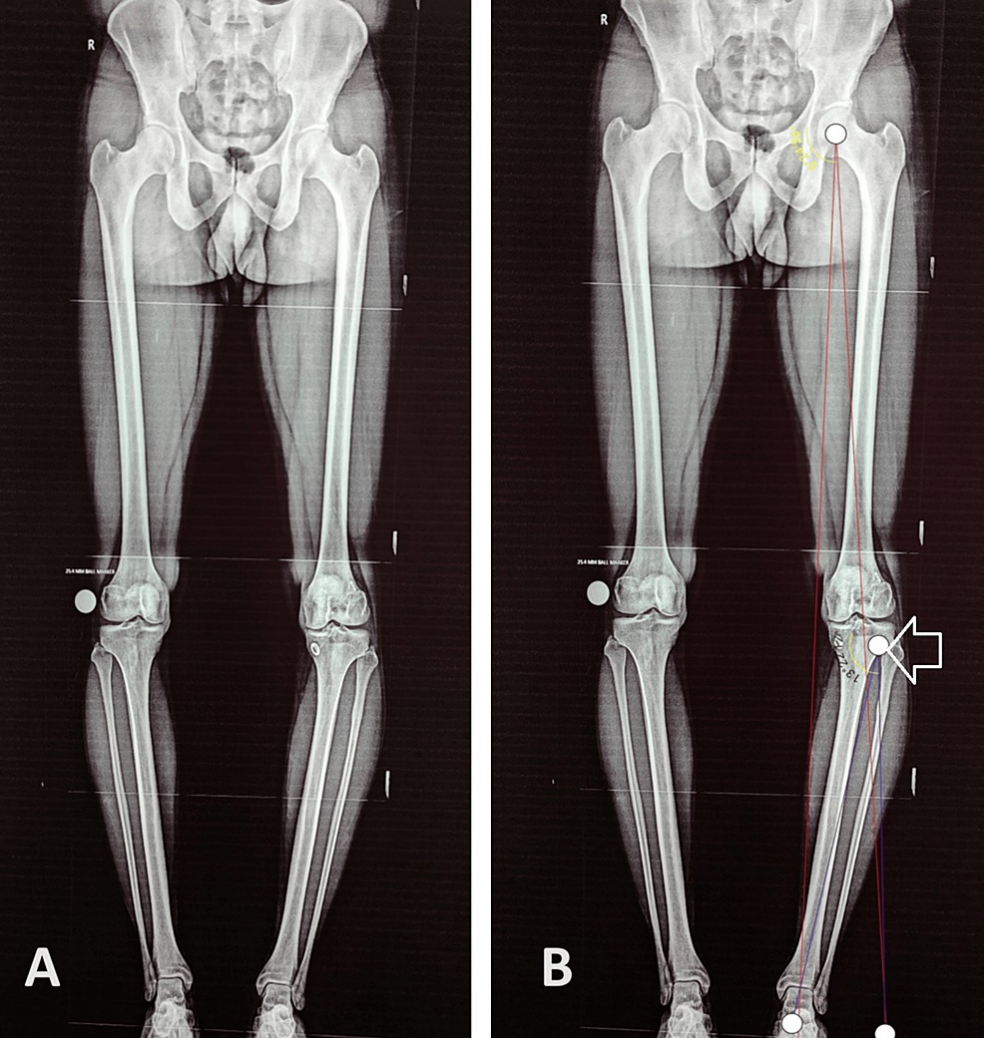
Standing scanogram of both lower limbs with patella facing forwards (A) Plain scanogram; (B) Using the Miniacci method, we calculated the amount of correction to be 13 degrees (marked by white arrow).

**Figure 2:**
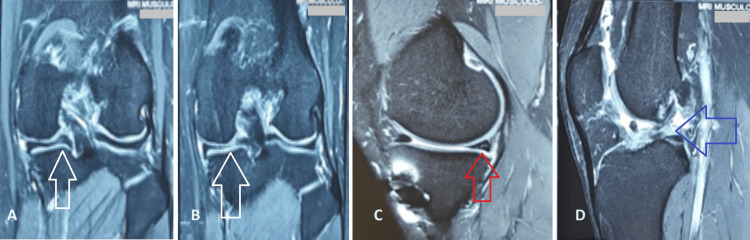
MRI sections of the left knee joint (A) Coronal section with white arrow pointing towards a signal in the posterior horn of medial meniscus; (B) Coronal section with white arrow pointing towards a signal in the posterior horn of medial meniscus; (C) Sagittal section with red arrow pointing towards a signal in the posterior horn of medial meniscus; (D) Sagittal section with blue arrow pointing towards the anterior cruciate ligament (ACL) tear.

On clinical examination, he had a varus alignment at both knees but walked without any thrust gait. There was distal tibia intortion bilaterally. He had no posterior tibial sag. He had a Lachman’s and Anterior Drawer test grade III positive. He had a significant pivot shift test positive. The McMurray test and Apley's test for both menisci were negative. Varus and valgus tests for collateral ligament competence were negative. The dial test was also negative. Based on clinical examination, we made a diagnosis of ACL graft rupture. To confirm our diagnosis, we ordered an MRI scan of the affected leg along with a weight-bearing full-length scanogram to assess the degree of varus deformity at the knee. Using the Miniacci technique [19], we got the degree of correction to be 13° to achieve the weight bearing passing through the center of the tibial spines (Figure 1). The MRI scans showed a complete tear of the ACL graft with a possible undersurface posterior horn medial meniscus tear (Figure 2). We got a CT scan to check the status of the tunnels and deemed that there was not any significant tunnel widening and a single-stage revision surgery could be done.

After reviewing the reports, we decided to go for a revision ACLR with a varus-correcting MOWHTO. All preoperative investigations were done, and the patient was taken up for surgery after anesthetic evaluation.

**Figure 3:**
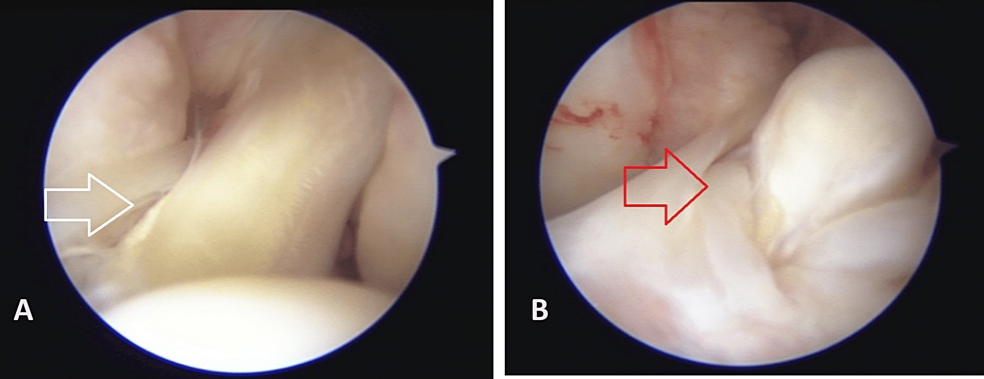
Diagnostic arthroscopy images (A) White arrow pointing towards friations seen in the anterior cruciate ligament (ACL) graft; (B) Red arrow pointing towards the tear in the ACL graft.

A diagnostic arthroscopic round of the knee was done and a tear in the ACL graft was diagnosed (Figure 3). The menisci had no tears, and the chondral cartilage was normal in all three compartments. Then the graft remnants were debrided using a shaver and we tried to locate the previous femoral and tibial tunnels. We felt that tunnels that were previously made were non-anatomical. The femoral tunnel was too high on the lateral femoral condyle. The tibial tunnel was oblique and was opening very proximal and medially in the tibial metaphyseal region. These non-anatomical tunnels and the varus alignment might have together resulted in a non-functioning ACL graft, which eventually tore.

**Figure 4:**
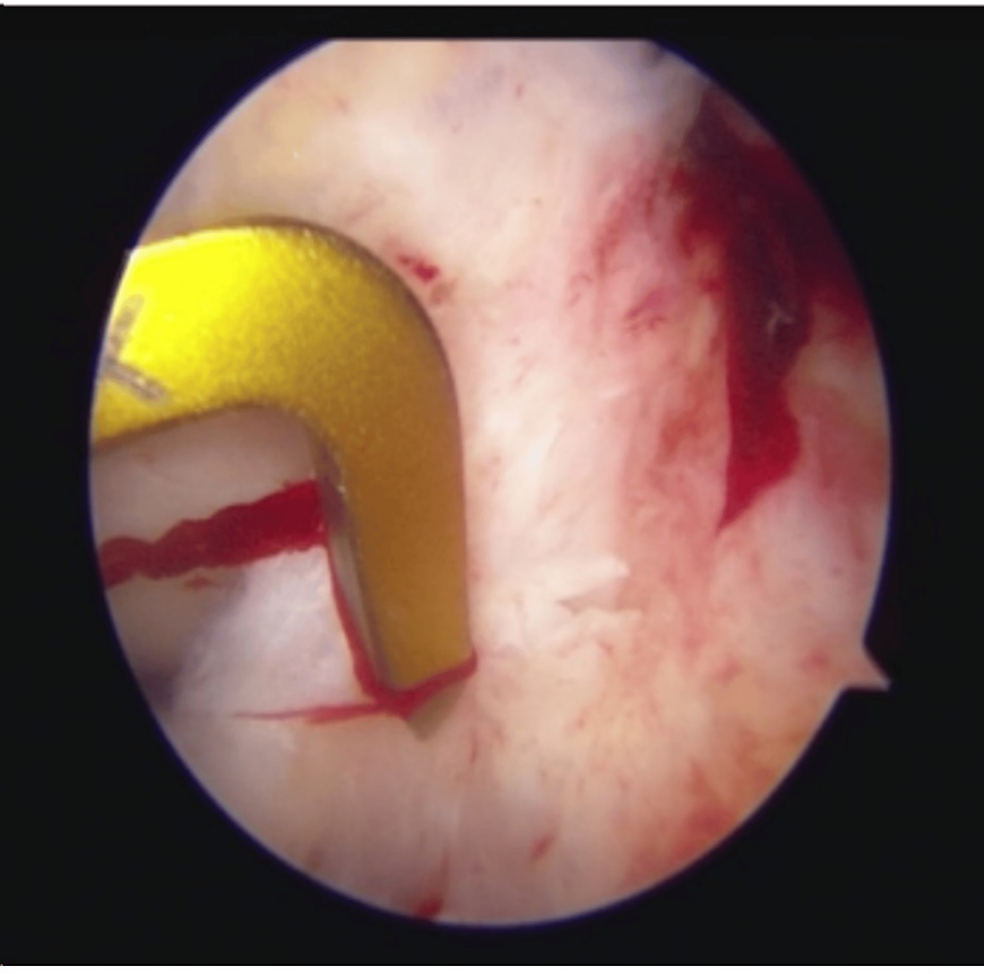
Tibial tunnel being marked with a zig

**Figure 5:**
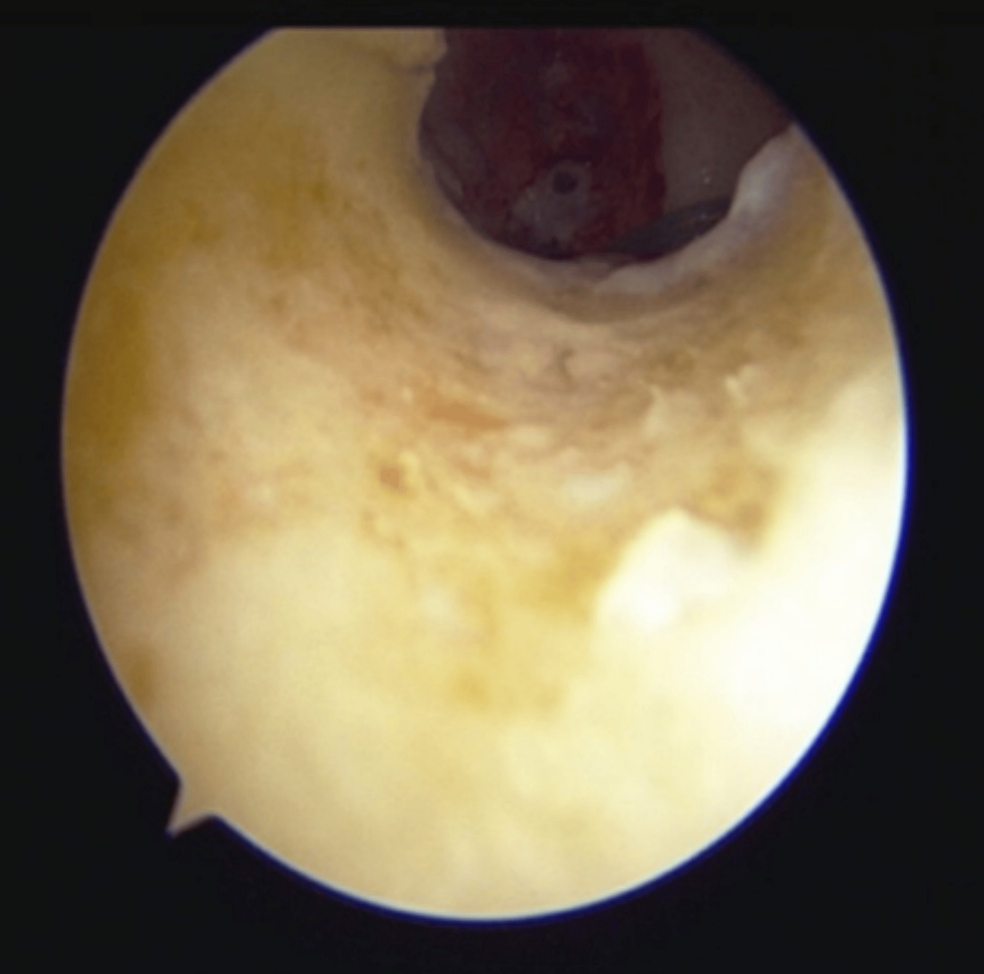
Arthroscopic view showing tibial tunnel cleared of all previous graft material

**Figure 6:**
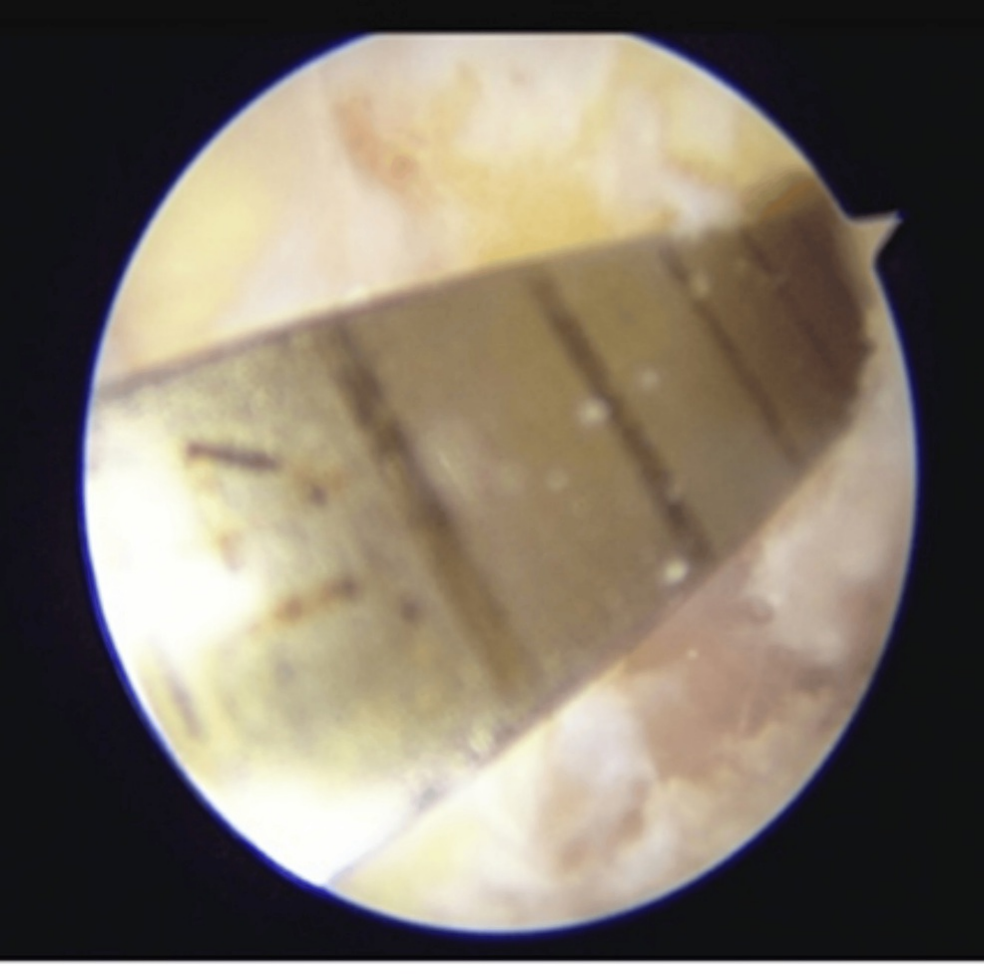
Arthroscopic view of femoral tunnel being prepared

The tibial tunnel was marked with the zig just medial to the anterior horn of the lateral meniscus (Figure 4). The tunnel was drilled to 9.5 mm while keeping the opening of the new tunnel on the tibial shin converging with the previous tunnel opening. The tunnel walls were cleared of previous graft materials and suture fibers using a curette and a shaver (Figure 5). A new femoral tunnel was marked, which was in a more anatomical position over the lateral femoral condyle. The femoral tunnel entry was marked just over the intercondylar ridge in the center of the footprints of native posterolateral and anteromedial fibers. The femoral tunnel was made through the transportal method and drilled up to 9.5 mm to a depth of 20 mm in the femoral condyle. The total femoral tunnel was 36 mm which was drilled using a 4.5 mm drill bit (Figure 6). Once the tunnel was drilled, we did skin marking of the lateral femoral condyle, Gerdys tubercle, and the head of the fibula. A 10 cm incision was made over the lateral aspect of the femur. A point was selected posterior and superior to the lateral epicondyle for fixation of the iliotibial (IT) band during the lateral extraarticular tenodesis. A guide pin was passed at this point in such a way that it did not interfere with the femoral tunnel. This was checked by checking the intraarticular view of the femoral tunnel while passing the guide pin.

**Figure 7:**
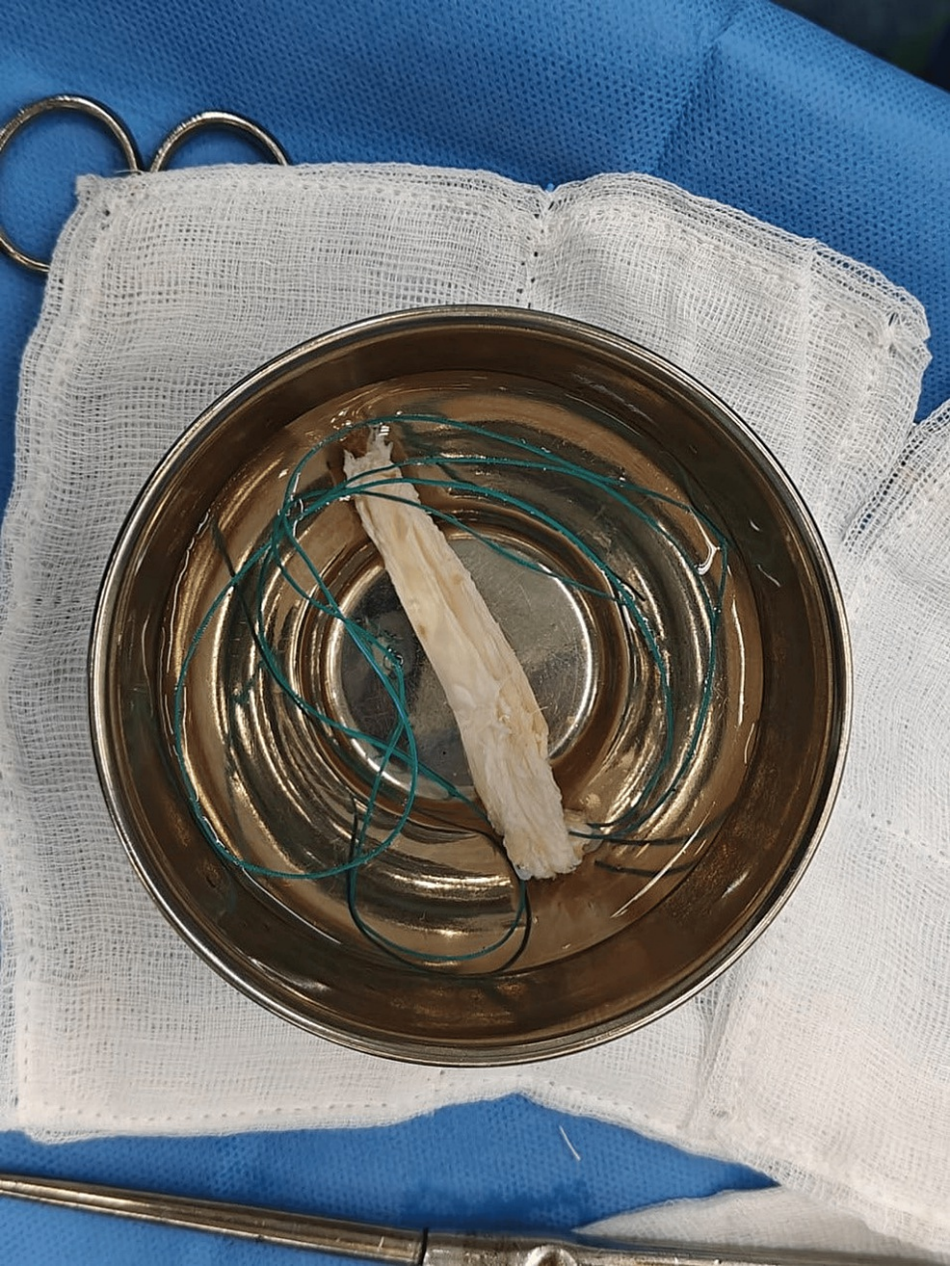
Quadriceps graft harvested and kept in antibiotic solution

Since the hamstring tendons were already utilized in the index surgery, we chose to go with a central quadriceps tendon autograft. We marked the patella and the femoral condyles, and we made a small 5 cm skin incision over the center of the quadriceps tendon. Tendon visibility was increased using removing the fat with a periosteum. Using a quadriceps graft harvester, we harvested a quadriceps graft of a thickness of 9 mm and a length of 70 mm (Figure 7). The graft was prepared using a quadriceps graft Arthrex TightRope® (Arthrex, Inc., Naples, Florida, United States) at the femoral end while the tibial end was prepared with No. 2 fiber wires. Once prepared, the graft was kept in an antibiotic solution till we proceeded with the further steps of the surgery.

Then we proceeded with the HTO. The site of the horizontal limb of the osteotomy medial to the tibial tuberosity and below the ACL tibial tunnel opening was marked such that the tibial tunnel remains completely in the proximal fragment of the osteotomy. Multiple drill holes were taken over the osteotomy site. We then passed a hinge pin at the lateral tibial cortex to protect the hinge. The osteotomy was completed with serial osteotomes passed from the medial tibial cortex to the hinge point about 10 mm from the lateral tibial cortex near the fibular head tip. We measured the depth till which the osteotomes penetrated the bone. Once the horizontal limb of the osteotomy was completed, we proceeded to make the vertical limb with the help of a saw. The vertical limb was made at an angle of 110^o ^to the horizontal limb. Once that was completed, we passed the spreader into the horizontal limb of the osteotomy up to the depth that was created by the osteotomes. Then we opened the spreader up to get a 12^o^ correction as was calculated on the preoperative scanogram.

At this stage, the alignment was checked with an alignment rod. However, we weren’t getting the alignment rod to pass from the center of the knee, hip, and ankle despite clinical valgus being seen at the level of the knee. We decided to stay with this amount of correction based on our preoperative calculations and clinical judgment. We measured the height of the medial opening with the help of a caliper and then the spreader was removed. A lamina spreader was placed at the most posterior aspect of the osteotomy and the osteotomy was opened till the caliper measurement and the spreader was locked in this position.

We then went ahead with fixing the osteotomy with the AO-DePuy Synthes TomoFix plate (Johnson & Johnson, New Brunswick, New Jersey, United States). However, unlike a primary alignment correction with ACLR, we could not place the tibial tunnel opening as per our choice. As a result, when we tried to put the plate, we realized that the previous tunnel had been placed by the earlier surgeon medial to what would have been ideal for the TomoFix plate placement. So, we had to place the tibial plate as posterior as posible on the medial surface of the tibia. Despite placing the tibial plate as was possible on the bone, the anterior edge of the plate was over the tunnel. However, there was enough space to pass the prepared graft through the tibial tunnel.

A guide pin was kept in the tibial tunnel, and we started fixing the TomoFix plate. Its level was confirmed to be below the level of the joint line on fluoroscopy. It was held in place by passing a pin through the sleeves of one of the proximal three screws. We proceeded with passing the proximal locking screws. After passing the posterior two screws of the appropriate length as measured on the depth gauge, we passed the drill bit through the locking sleeve of the anteriormost proximal hole. However, it hit the guide pin placed in the tibial tunnel. So, we didn’t put a screw through the anterior screw hole, as it would have caused difficulty in graft passage through the tunnel. We then placed a cortical screw in the combi hole of the distal fragment to get compression at the lateral cortical hinge.

**Figure 8:**
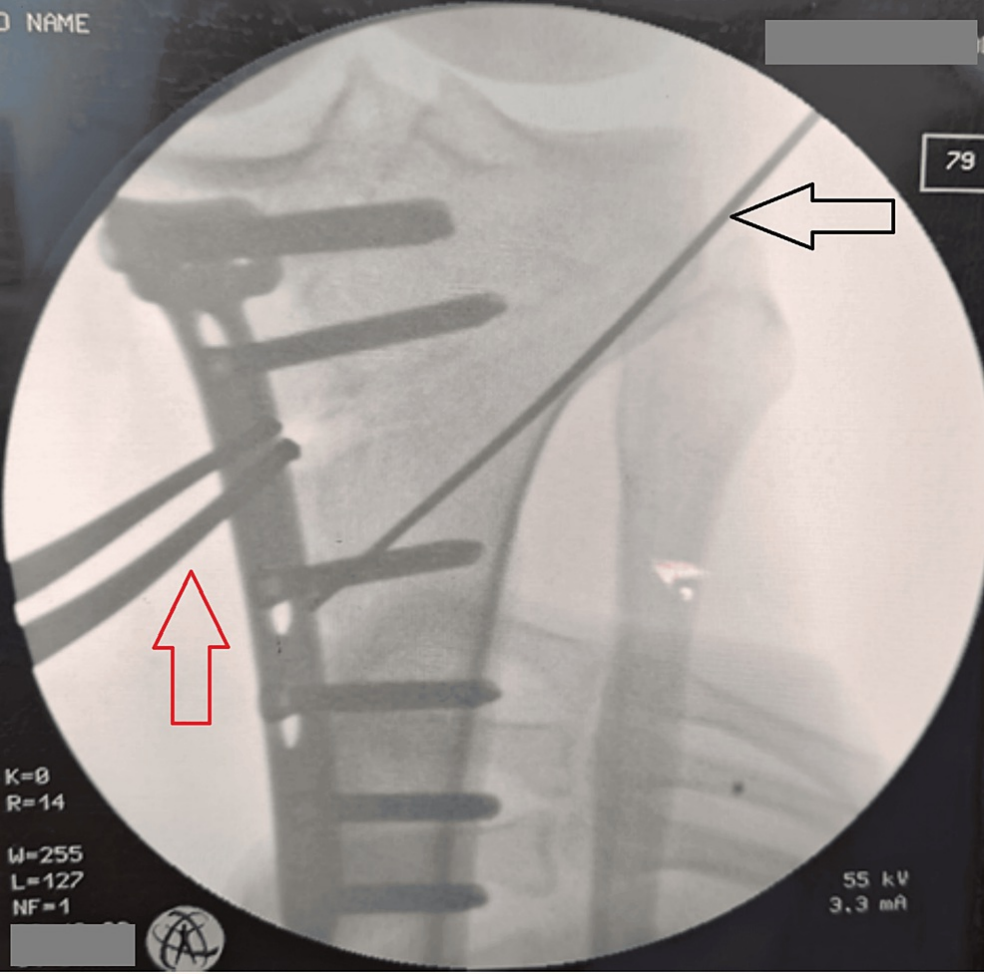
Intraoperative fluroscopic image after fixation of osteotomy with TomoFix plate Red arrow pointing towards the lamina spreader keeping the osteotomy open while black arrow pointing towards the lateral cortical hinge pin.

Once plate compression was achieved, the bush screws were removed from the D-hole and the distal locking hole. Locking screws of appropriate sizes were passed in the remaining locking holes of the plate in the distal fragment. While keeping the guide pin in the tibial tunnel, we drilled the D-hole with a drill bit so as to make sure the screw didn’t enter the tibial tunnel. A locking screw of appropriate length such that it didn’t pass into the osteotomy site was then passed through the D-hole. The cortical screw was then replaced by a locking screw of an appropriate length in the locking hole of the combi hole. The final position of the plate and osteotomy were checked on fluoroscopy (Figure 8).

A Beath pin was then passed through the femoral tunnel under arthroscopic guidance with the knee in hyperflexion. A shuttle Ethibond no. 5 suture (Johnson & Johnson) was passed, which was then taken into the tibial tunnel with a grasper. We removed the graft from the vancomycin antibiotic solution and trimmed off the loose ends. The graft was dried, and two points were marked at the femoral end of the graft with a sterile marker pen. One was on the TightRope at 36 mm from the button to know the length of suture that has passed through so that the button can be flipped onto the femoral condyle. Another was marked on the femoral end of the graft at 20 mm because this was the amount of graft that had to be inserted into the femoral tunnel. The graft was passed through the tunnels using the shuttle Ethibond suture under arthroscopic view. The TightRope button was flipped over the femoral condyle using the flip suture and checked by giving traction. Once confirmed that it had flipped, the graft was pulled inside the femoral tunnel till the marked point.

Keeping traction on the tibial end of the graft, we did serial cycling of the ACL graft. Then the knee was kept at 30 degrees of flexion and the graft was at the tibial tunnel mouth. We decided to go for a compressive fixation at the tibial end. A guide pin for the screw was placed through the tibial tunnel such that it was anterolateral to that graft and the screw would push the graft medial and posterior and help in preventing anterior and lateral impingement. The guide wire was held intraarticularly with an artery forceps so that it did not change position while passing the screw. The knee was taken into full extension and tibial side fixation was done with a 10 x 30 mm MILAGRO™ Advance Interference Screw (Johnson & Johnson). Impingement was checked for in both flexion and extension. Intercondylar osteophytes were removed and a minimum Notchplasty was performed to prevent impingement in the future.

**Figure 9:**
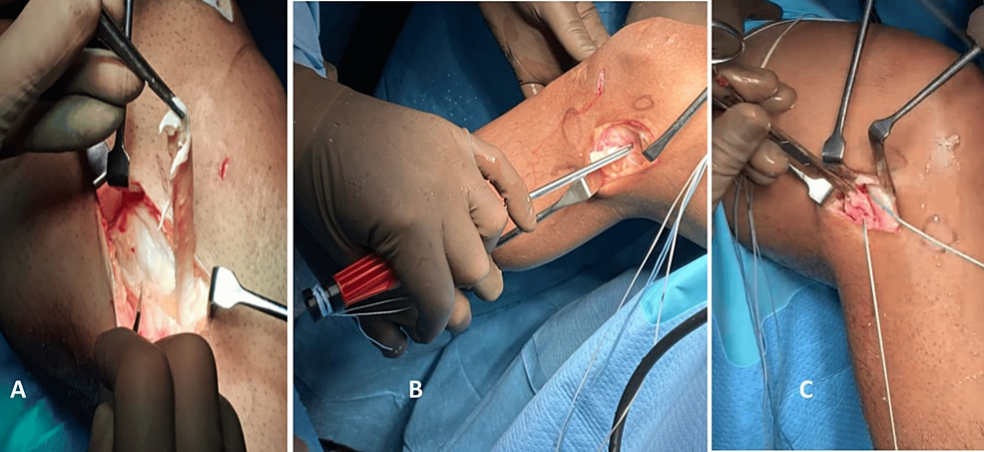
Lateral extra-articular tenodesis (A) Tensor fasciae latae strip (TFL) being released; (B) All-suture anchor being inserted posterior and superior to the lateral epicondyle; (C) TFL strip fixed with anchor.

Once this was completed, we proceeded to finish the lateral extraarticular tenodesis. A 15 x 70 mm strip of tensor fasciae latae (TFL) was cut above the lower one-third of its breath. The strip was attached distally and released proximally. The lateral collateral ligament (LCL) was identified, and a tunnel was prepared underneath it. The strip of TFL was passed underneath it. The guide pin which was passed earlier posterior and superior to the lateral epicondyle was removed and a 2.3 mm double-loaded all-suture anchor was passed in the same direction. The TFL strip was tied to the suture anchor at this point with the knee in 30^o^ of flexion and neutral rotation (Figure 9). The extra length of the strip was tied onto itself using Vicryl No. 1 (Johnson & Johnson).

**Figure 10:**
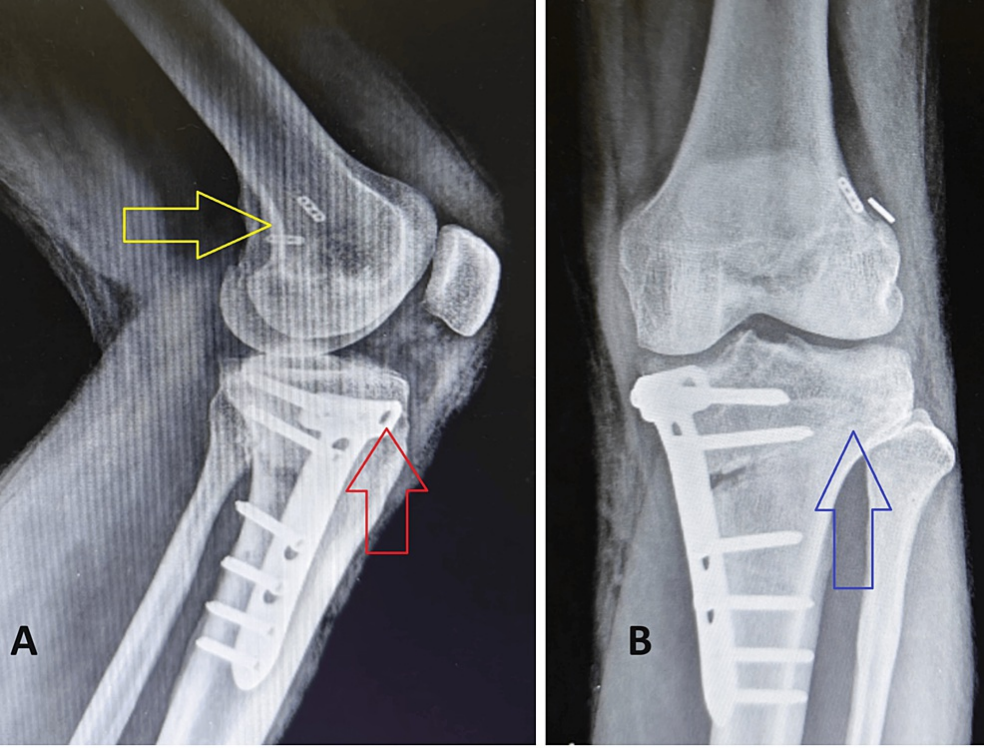
Postoperative radiographs of the left knee joint at six weeks after surgery (A) Lateral radiograph with yellow arrow pointing towards femoral fixation buttons of the current and initial surgery, red arrow pointing towards anterior proximal hole being left open without a screw; (B) Anteroposterior radiograph with blue arrow pointing towards compressing at lateral hinge point of osteotomy.

**Figure 11:**
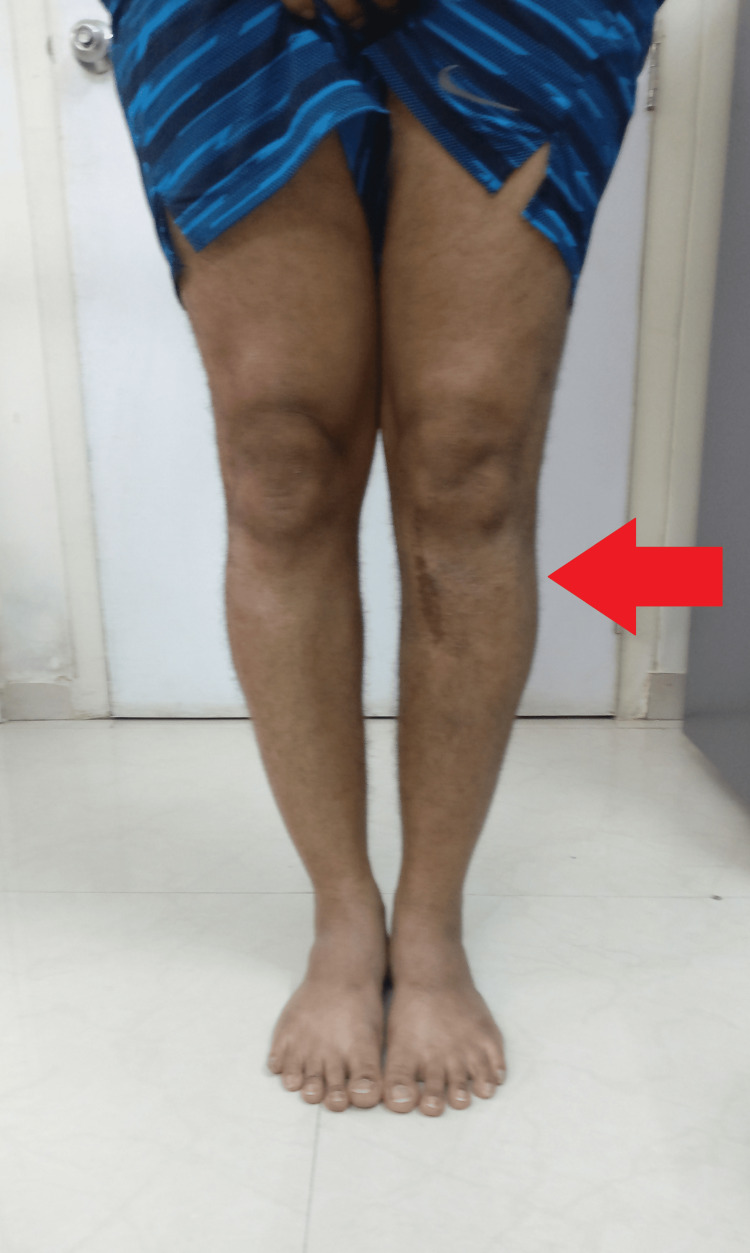
Clinical picture at six months after the surgery Red arrow pointing towards significantly reduced varus in the left knee as compared to the right knee.

A thorough wash was given both intraarticularly and over the extraarticular incisions. Closure was done in layers and sterile dressing was done. The leg was put into a long knee brace. Isometric quadriceps and hamstring exercises were initiated immediately post-surgery. Knee bending up to 90^o^ was started after 48 hours of surgery. Partial weight-bearing mobilization was started after suture removal on the 14th day and, by six weeks, full weight-bearing mobilization was started. A radiograph taken at this stage showed good position of the implant and uniting osteotomy (Figure 10). Clinically varus was improved at the knee joint (Figure 11).

Knee bending beyond 90^o^ and isotonic strengthening exercises started after six weeks. Proprioception and balance exercises were initiated. Full range was achieved by 12 weeks post surgery after which gradual strengthening exercises and jogging and running were initiated. By six months post surgery, the patient had gained 90% of the muscle strength as compared to the opposite side (Figure 11). He was started on back-to-sports and injury prevention protocol at six months under the care of our physiotherapist.

## Discussion

In this case report, we are trying to highlight some important technical points and problems that we can encounter during a ACLR with a varus correction MOWTHO surgery in a single sitting. We have highlighted our observations in Table 1.

**Table 1:** Technical points, rationale, and problems encountered. ACL: anterior cruciate ligament

Surgical Step	Technical Tip	Rationale	Problems Encountered
Graft Choice	Soft tissue autograft like hamstring graft, central quadriceps tendon graft, or a peroneus longus graft as per availability	Since multiple soft tissue graft sites are available for graft harvest in revision surgeries. We also avoid the graft tunnel mismatch encountered with a bone patellar tendon graft.	In some obese individuals, central quadriceps tendon graft might have been utilized for the index surgery and hamstring and peroneus tendon grafts might not be thick enough to fill the femoral and tibial tunnels. Thicker grafts may cause impingement and may need added Notchplasty.
Femoral Tunnel Drilling	Search for the old femoral tunnel and its placement. If non anatomical in position go for new tunnel placement.	This avoids stress and hyperflexion of the knee which has risk of loss of correction if HTO is done before the femoral drilling.	Sometimes you might find it difficult to find the femoral tunnel. The non-anatomical tunnel placement might be such that it would be difficult to place an anatomical tunnel opening without causing significant widening of the femoral tunnel. Difficult to find a tunnel for lateral extraarticular tenodesis in small size femur condyles.
Tibial Tunnel Drilling	Search for the old tibial tunnel and check for its position with respect to the site of osteotomy. Make the tibial tunnel with the zig in such a way that the metaphyseal tunnel opening is above the site of osteotomy and is more anterior in placement so as to allow enough space for plate placement. Clear all the graft material from the walls of the tunnel with a curette and shaver.	By making the tibial tunnel we can then mark the osteotomy site and plate positioning as per the position of the tibial tunnel. Clearing the tunnel walls allows for smooth passage of the new graft through the tunnel as well as incorporation of Sharpey's fibers into the new graft.	Sometimes if we try to make a new tunnel it might converse with the old tunnel and cause significant tunnel widening. Try to use the old tunnel or make divergent tunnels as far as possible.
Tunnel Sizing	Usually, we go for a thicker graft than used for the index surgery. Preoperative CT should be done to see if there is significant widening beyond 15 mm.	We prefer a thicker graft for better stability. If there is significant tunnel widening preoperatively then we need to go for two-stage surgery. In thefirst stage, we go for bone grafting of the tunnels. After eight weeks, we go for the second stage ACL reconstruction.	Graft and tunnel size mismatch might take place.
High Tibial Osteotomy	Protect the patellar tendon with a retractor and the posterior neurovascular structures with a radiolucent Hohmanns retractor. The lateral cortical hinge must be protected by passing a hinge pin at the lateral tibial cortex. The osteotomes should be advanced up to 10 mm distance from the lateral tibial cortex while being directed toward the tip of the fibula. Make the horizontal limb of the osteotomy at 110 degrees to the vertical limb using a saw. Once the HTO is finished, move the handle of the last osteotome distally or use a mild valgus force to assess the osteotomy site's mobility. Make sure the anterior and posterior cortices have been completely cut if the osteotomy does not open. Generally speaking, the plate is positioned more posteriorly when compared to HTO for isolated medial OA. The lamina spreader is paced on the posterior aspect of the vertical osteotomy to open up the osteotomy. Once the correction is achieved, we check the correction using an alignment rod. Proximal screws are placed while keeping a pin in the tibial tunnel.	Do not injure these structures. Using a radiolucent Homanns retractor helps using in making the osteotomy cut while protecting the posterior structures. Prevent the osteotomy from migrating intra-articularly or causing lateral hinge rupture, which could result in mediolateral displacement of the pieces. It helps in correcting the deformity in two planes. Avert distal or intra-articular osteotomy migration. This leaves more room on the anteromedial tibia for the tibial tunnel opening. This reduces the posterior tibial slope which protects the ACL graft from tension. Clinical alignment is also important. Checking the alignment with the alignment rod helps us confirm the correction is correct. Usually, the anterior screw might communicate with the tibial tunnel and this can be checked if the pin is disturbed while drilling the screw hole. In such a case that screw should be left.	Distal pulses must be checked post-surgery for vascular status. The lateral hinge may break and the alignment might be lost. We cannot mobilise the patient postoperatively if the hinge breaks If osteotomy is opened anteriorly then we increase the tibial slope and cause failure of the ACL reconstruction. The tibial tunnel opening of the index surgery might cause difficulty in the placement of the osteotomy and the plate placement. The tibial tunnel may communicate with the osteotomy Proximal screws if passed after graft placement may cause disruption of the tibial tunnel graft. Alignment rod might not show an adequate correction as some amount of distal deformity might exist in the tibia. This should be accounted for while checking the post-osteotomy correction of alignment.
Lateral Extraarticular Tenodesis	Check the femoral tunnel intraarticularly while making the tunnel for lateral extraarticular tenodesis.	This is to make sure that the two tunnels do not converge.	In small femoral condyles, it is difficult to avoid convergence.

There are different viewpoints on how individuals with medial compartment OA and ACL deficit should be treated. For patients and surgeons alike, combined HTO and ACLR is a challenging treatment. O'Neill et al. originally reported simultaneous combined ACLR and closed wedge HTO for young patients with medial joint pain and instability brought on by medial compartment OA and persistent ACL insufficiency. Every patient had less discomfort and a resolution to their instability. They concluded that the simultaneous procedure produced better short-term results and a reduced rate of complications [15]. According to Bonin et al., concurrent closed or open wedge HTO and combined ACLR produced acceptable long-term outcomes [16].

However, young patients may have positive outcomes if there is meticulous patient selection. Noyes et al., in their 2000 study, treated 41 patients who had a varus lower limb alignment along with ACL insufficiency [17]. Most of the patients in his study didn’t undergo a single stage surgery and initially underwent a high tibial osteotomy followed by an ACL reconstruction surgery a mean eight months post the realignment surgery. They had a mean follow-up period of 4.5 years. The Cincinnati knee rating score significantly improved in the patients from 63 points to 82; 85% of patients didn’t have repeat instability, 71% had a reduction in pain, and 66% returned to recreational activities without symptoms. This study showed that alignment correction with ACLR has good clinical outcomes [17]. However, unlike the current case, most cases in this study underwent a two-stage procedure.

Yasushi et al. in their case series of four cases performed a simultaneous arthroscopic ACLR with an OWHTO using the TomoFix fixation plate and hydroxyapatite wedges. Patients started full weight-bearing mobilization by four weeks post surgery. They performed a diagnostic arthroscopy at the time of plate removal and saw a cyclops-like lesion and partial tears between the ACL graft and intercondylar notch. So, they had to perform a Notchplasty in these patients and therefore advocated doing a prophylactic intercondylar Notchplasty in the initial surgery to prevent impingement and cyclops formation [18]. We performed a prophylactic Notchplasty in the current case since we had used a thicker quadriceps graft.

Bonasia et al., in their technical note for performing arthroscopic ACLR with MOWHTO, described the surgical steps and technical points that need to be known while performing this single-stage procedure [13]. Some of these technical points were kept in mind while performing our surgery and we added to them based on our experience.

Li et al., in their systemic review of simultaneous arthroscopic ACLR with HTO in young patients, reviewed 11 level III and II publications, and 85.7% of the patients after surgery had Grade A or B stability as per the International Knee Documentation Committee evaluation with the mean side-to-side difference as determined by KT-1000 being 2.4 mm [20]. There was an apparent relief tendency in medial compartment osteoarthritis. All subjective assessments demonstrated improvement, independent of the scoring method, and the majority of the individuals resumed their recreational sports. A mean value of 7.13° was obtained after correcting all cases of varus malalignment. They concluded that combined HTO and ACLR was a salvage procedure for young patients who were physically active because of its satisfactory restoration of anterior stability, alleviation of medial compartment osteoarthritis, improvement of subjective evaluations, and predictable return to recreational sports [20].

## Conclusions

In young patients with a varus alignment with simultaneous instability due to ACL instability, we could consider a single-stage arthroscopic ACLR with MOWHTO as it gives excellent results. The patient has relief from his instability symptoms while the realignment surgery protects the new graft from excessive tension and subsequent failure. In revision surgeries, we prefer a thick autograft as well as augmenting the reconstruction with an extraarticular procedure like lateral extraarticular tenodesis. This is a technically demanding procedure to be performed in one stage and good preoperative planning and knowledge of technical surgical aspects must be kept in mind when the surgeon is presented with such a case.

## References

[1] Lien-Iversen T, Morgan DB, Jensen C, Risberg MA, Engebretsen L, Viberg B (2020). Does surgery reduce knee osteoarthritis, meniscal injury and subsequent complications compared with non-surgery after ACL rupture with at least 10 years follow-up? A systematic review and meta-analysis. Br J Sports Med.

[2] Buller LT, Best MJ, Baraga MG, Kaplan LD (2015). Trends in anterior cruciate ligament reconstruction in the United States. Orthop J Sports Med.

[3] Noyes FR, Barber-Westin SD (2001). Revision anterior cruciate surgery with use of bone-patellar tendon-bone autogenous grafts. J Bone Joint Surg Am.

[4] Lind M, Menhert F, Pedersen AB (2009). The first results from the Danish ACL reconstruction registry: epidemiologic and 2 year follow-up results from 5,818 knee ligament reconstructions. Knee Surg Sports Traumatol Arthrosc.

[5] Maletis GB, Granan LP, Inacio MC, Funahashi TT, Engebretsen L (2011). Comparison of community-based ACL reconstruction registries in the U.S. and Norway. J Bone Joint Surg Am.

[6] Nagaraj R, Kumar MN (2019). Revision anterior cruciate ligament reconstruction in the nonathlete population. Indian J Orthop.

[7] Samuelsen BT, Webster KE, Johnson NR, Hewett TE, Krych AJ (2017). Hamstring autograft versus patellar tendon autograft for ACL reconstruction: is there a difference in graft failure rate? A meta-analysis of 47,613 patients. Clin Orthop Relat Res.

[8] Rossi R, Bonasia DE, Amendola A (2011). The role of high tibial osteotomy in the varus knee. J Am Acad Orthop Surg.

[9] Dejour H, Neyret P, Boileau P, Donell ST (1994). Anterior cruciate reconstruction combined with valgus tibial osteotomy. Clin Orthop Relat Res.

[10] Amendola A, Bonasia DE (2010). Results of high tibial osteotomy: review of the literature. Int Orthop.

[11] Duivenvoorden T, Brouwer RW, Baan A, Bos PK, Reijman M, Bierma-Zeinstra SM, Verhaar JA (2014). Comparison of closing-wedge and opening-wedge high tibial osteotomy for medial compartment osteoarthritis of the knee: a randomized controlled trial with a six-year follow-up. J Bone Joint Surg Am.

[12] Schuster P, Geßlein M, Schlumberger M, Mayer P, Richter J (2018). The influence of tibial slope on the graft in combined high tibial osteotomy and anterior cruciate ligament reconstruction. Knee.

[13] Bonasia DE, Dettoni F, Palazzolo A, Rossi R (2017). Opening wedge high tibial osteotomy and anterior cruciate ligament reconstruction or revision. Arthrosc Tech.

[14] Noyes FR, Goebel SX, West J (2005). Opening wedge tibial osteotomy: the 3-triangle method to correct axial alignment and tibial slope. Am J Sports Med.

[15] O'Neill DF, James SL (1992). Valgus osteotomy with anterior cruciate ligament laxity. Clin Orthop Relat Res.

[16] Bonin N, Ait Si Selmi T, Donell ST, Dejour H, Neyret P (2004). Anterior cruciate reconstruction combined with valgus upper tibial osteotomy: 12 years follow-up. Knee.

[17] Noyes FR, Barber-Westin SD, Hewett TE (2000). High tibial osteotomy and ligament reconstruction for varus angulated anterior cruciate ligament-deficient knees. Am J Sports Med.

[18] Akamatsu Y, Mitsugi N, Taki N, Takeuchi R, Saito T (2010). Simultaneous anterior cruciate ligament reconstruction and opening wedge high tibial osteotomy: report of four cases. Knee.

[19] Miniaci A, Ballmer FT, Ballmer PM, Jakob RP (1989). Proximal tibial osteotomy. A new fixation device. Clin Orthop.

[20] Li Y, Zhang H, Zhang J, Li X, Song G, Feng H (2015). Clinical outcome of simultaneous high tibial osteotomy and anterior cruciate ligament reconstruction for medial compartment osteoarthritis in young patients with anterior cruciate ligament-deficient knees: a systematic review. Arthroscopy.

